# Detection of Glutamate Decarboxylase Antibodies and Simultaneous Multi-Molecular Translocation Exploration by Glass Nanopores

**DOI:** 10.3390/bios14050255

**Published:** 2024-05-17

**Authors:** Chongxin Tao, Yun Bai, Jiang Chen, Jing Lu, Yan Bi, Jian Li

**Affiliations:** 1 Key Laboratory of DGHD, MOE, School of Life Science and Technology, Southeast University, Nanjing 210096, China; 2 Department of Endocrinology, Nanjing Drum Tower Hospital, Affiliated Hospital of Medical School, Nanjing University, Nanjing 210008, China

**Keywords:** glass nanopore, single molecule detection, glutamate decarboxylase antibody, multi-molecular translocation, type I diabetes

## Abstract

Glutamic acid decarboxylase antibody (GADAb) has emerged as a significant biomarker for clinical diagnosis and prognosis in type 1 diabetes (T1D). In this study, we investigated the potential utilization of glass capillary solid-state nanopores as a cost-effective and easily preparable platform for the detection of individual antigens, antibodies, and antigen-antibody complexes without necessitating any modifications to the nanopores. Our findings revealed notable characteristic variations in the translocation events of glutamic acid decarboxylase (GAD65) through nanopores under different voltage conditions, discovered that anomalous phenomenon of protein translocation events increasing with voltage may potentially be caused by the crowding of multiple proteins in the nanopores, and demonstrated that there are multiple components in the polyclonal antibodies (GADAb-poly). Furthermore, we achieved successful differentiation between GAD65, GADAb, and GADAb-GAD65 complexes. These results offer promising prospects for the development of a rapid and reliable GADAb detection method, which holds the potential to be applied in patient serum samples, thereby facilitating a label-free, cost-effective, and early diagnosis of type I diabetes.

## 1. Introduction

Diabetes mellitus is a very common endocrine disease in clinical practice, which is mainly divided into type I diabetes (T1DM), characterized by pancreatic β-cell damage, and type II diabetes (T2DM), characterized by insulin resistance [1]. Due to the large differences in the disease process and treatment methods between type I and II diabetes, early diagnosis and classification are of great significance for their treatment and prognosis. As an autoimmune disease, the diagnosis and classification of type I diabetes depend on a series of autoimmune antibodies against the secreted proteins of pancreatic β-cells. Among them, the autoimmune antibodies of glutamic acid decarboxylase (GAD65), are one of the most sensitive and specific clinical markers, which have been used in combination with several other autoimmune antibodies (like ICA and IAA) as the diagnostic criteria for type I diabetes in clinical practice [2,3]. Currently, the detection of GADAb in clinical practice mainly relies on several traditional methods, including ELISA [4,5], RIA [6], and immunofluorescence [7], which exhibit excellent reliability and are widely used for the quantitative detection of specific antigens and antibodies. Apart from these gold standard methods, single-molecule detection technology has gradually become a hotspot in the field of biological analysis due to its high sensitivity, as well as high-throughput advantages. However, the reported single-molecule immunofluorescence detection and single-molecule electrochemical immunoassay methods still rely on various biomarkers [8,9], which limits the promotion of single-molecule immunoassay technology in applications.

Nanopore technology provides a label-free, high-throughput single-molecule detection method at the nanometer scale [10,11,12]. Its detection principle is based on the concept of the Coulter counter [13]. When a stable external electric field is applied on both sides of the nanopore, the ions in the electrolyte on both sides of the nanopore move directionally and produce a weak but relatively stable ion current. When charged analyte biomolecules pass through the nanopore under the driving force of the electric field, they will interfere with the ion current and produce detectable pulse signals. According to the characteristics of signal amplitude, duration, and event frequency, it can be inferred that the detected biomolecules have certain structural, size, and charge states [14,15,16,17].

In previous studies, biological nanopores have been widely used in DNA sequencing [18], microRNA detection [19], and analysis of oligopeptides and proteins [20]. Research on antigen-antibody interactions has also been reported [21], but the lower stability and fixed pore size limit the development of biological nanopores in practical immunoassay applications. At the same time, solid-state nanopores have more stable physicochemical properties and adjustable sizes compared to biological nanopores, which greatly expand the application potential of solid-state nanopores in the field of single-molecule detection [22,23]. Glass capillary solid-state nanopores, as members of solid-state nanopores, have advantages of high stiffness, low cost, and reproducible size and shape. Their related applications in protein recognition have been reported many times [24,25,26]. In these applications, the differences in protein size and charge state are the keys to distinguishing them.

Therefore, in this study, quartz glass nanopores were used to investigate the translocation behavior of glutamate decarboxylase (GAD65), glutamate decarboxylase antibodies (GADAb), and the antigen-antibody complex (GAD65-GADAb complex). Due to the differences in charge and molecular diameter among the three molecules, there are differences in the characteristics of translocation signals. By distinguishing the translocation characteristics of the immune complexes, it provides a potential application for the development of new single-molecule detection methods for GAD65 and GADAb in clinical practice, which is cheap, sensitive, and high-throughput.

## 2. Materials and Methods

### 2.1. Chemical and Reagents

GAD65 was purchased from Abcam (Cambridge, UK) and GADAb (both monoclonal and polyclonal) were purchased from Sigma-Aldrich (Shanghai, China). Tris-HCl (pH = 8.0) was produced by Solarbio (Beijing, China). Both KCl and ethanol were purchased from Sinopharm (Shanghai, China). Electrolyte for translocation through nanopore was 1 M KCl solution contain 10 mM Tris-HCl (pH = 8.0). All solutions were prepared with Milli-Q water (18 MΩ·cm resistivity) from a Millipore system (Merck, Darmstadt, Germany).

### 2.2. Device Construction

**Figure 1:**
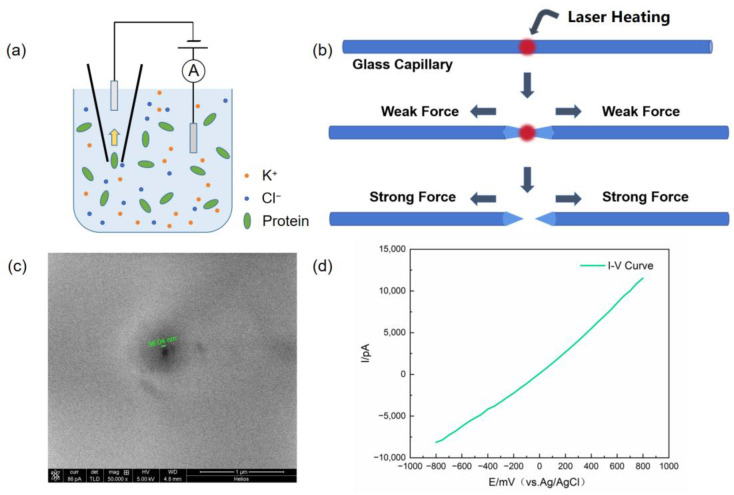
Fabrication of glass nanopores and device structure. (**a**) Schematic diagram of glass nanopore device for detecting proteins in ionic solutions. (**b**) Schematic of glass nanopore fabrication. (**c**) Characterization of glass nanopore size using scanning electron microscopy (SEM). (**d**) Characterization of glass nanopore size using I-V curve approximation.

Two Ag/AgCl electrodes were inserted into the electrochemical cell filled with electrolyte (1 M KCl, 10 mM Tris-HCl, pH = 8.0). During the detection of protein molecules, the protein molecules were added to the electrolyte solution, with the glass nanopore fixed to the negative electrode and the positive electrode added outside the nanopore (as shown in Figure 1a). The other ends of the electrodes were connected to the Axopatch 700B patch clamp amplifier (Molecular Devices, San Jose, CA, USA). The signals were digitized using the Axon Digidata 1550A digital-to-analog converter (Molecular Devices, San Jose, CA, USA) and viewed using Clampfit software (Version 10.5.2.6).

### 2.3. Fabrication of Glass Nanopores and Characterization

Quartz glass capillaries used in the experiments (QF100-70-10, Sutter Instrument Co., Novato, CA, USA) have an outer diameter of 1 mm, an inner diameter of 0.7 mm, and a length of 7.5 cm. Before use, the glass capillaries were ultrasonically cleaned in ethanol and pure water for 15 min, and the liquid residue on the tube walls was removed using a nitrogen gas stream. The capillaries were then pulled into nanopores using a CO_2_ laser capillary puller (model P-2000, Sutter Instruments Co., Novato, CA, USA) with the parameters shown in Table 1:

The glass nanopore was electrochemically characterized in electrolyte (1 M KCl, 10 mM Tris-HCl, pH = 8.0) using cyclic voltammetry. The ion current was measured at intervals of 100 mV in the range of −500 mV to +500 mV, and an I-V scan curve was plotted accordingly. The conductance value of the nanopore was determined using the slope of the curve, and the diameter of the glass nanopore was calculated. Subsequently, the morphological characteristics and diameter of the glass nanopore were characterized using scanning electron microscopy (SEM) to verify the accuracy of the nanopore diameter estimation obtained using electrochemical methods.

### 2.4. Protein Translocation

The glutamate decarboxylase (GAD65, Abcam ab206646) and glutamate decarboxylase monoclonal antibody (GADAb-mono, Sigma-Aldrich G1166, derived from the GAD-6 hybridoma produced by the fusion of mouse myeloma cells and splenocytes from a mouse immunized with purified rat brain GAD) were diluted to 1540 pmol/L and 667 pmol/L, respectively, in electrolyte buffer (1 M KCl, 10 mM Tris-HCl, pH = 8.0). The glutamate decarboxylase polyclonal antibody (GADAb-poly, Sigma-Aldrich G5163, produced in rabbits using a synthetic peptide KDIDFLIEEIERLGQDL corresponding to the C-terminal region of GAD 67 of human origin as immunogen) was diluted to 10,000 times of its original concentration using the buffer. The diluted samples were injected into the glass nanopore using a microinjector, while the electrochemical cell was filled with electrolyte.

The ionic currents were measured with Ag/AgCl electrodes inserted in buffer solution and recorded with the amplifier Axopatch 200B (Molecular Devices, San Jose, CA, USA) in voltage-clamp mode using a low-pass Bessel filter of 5 kHz. The signals were digitized with DigiData 1550s digitizer at 100 kHz and viewed with Clampfit 10.2 software. The protein translocation events were recorded, and the event features were extracted using the Transalyzer analysis package (Version RC1b) based on Matlab [27]. The amplitude, duration time, and baseline data of each translocation event were imported into Origin software (Version 2023b) to generate frequency distribution histograms and fitted curves for the basic characteristics of the translocation events under various conditions. The different characteristics of the protein translocation events were investigated by varying the applied voltage.

### 2.5. Detection of Immune Complex

The antigen and antibody (monoclonal) proteins mentioned in the previous steps were mixed in equal volumes at their respective concentrations and incubated at room temperature for 30 min. The resulting immune complexes were injected into the glass nanopore using the microinjector. The translocation currents generated by the immune complexes passing through the nanopore were measured and recorded using the device described earlier. The amplitude size, duration time, and baseline data of each translocation event were plotted as translocation time and ΔI/I_0_ frequency distribution histograms. The average effective molecular diameter of the immune complexes was calculated based on the statistical results and compared with the molecular diameter of the monomer antigen or antibody to distinguish between antigens, antibodies, and their immune complexes formed by binding.

### 2.6. Statistical Analysis Methods

The general statistical analysis methods for translocation signals include plotting scatter plots of duration time versus signal amplitude, histograms of duration time–frequency distribution, and histograms of signal amplitude of blockage (ΔI/I_0_) frequency distribution. Specifically, the raw data are processed using the Transalyzer analysis package (Version RC1b) and then imported into the Origin software (Version 2023b) to generate these three types of statistical analysis graphs. In the histogram of frequency distribution, the average duration time and the amplitude of blockage (ΔI/I_0_) can be obtained by fitting a Gaussian curve and determining its mean value, representing the average state of all signals. The equation for fitting the Gaussian curve is as follows:

(1) $$y=y_{0}+\frac{A}{w\sqrt{\pi /2}}e^{-2\frac{({x-x_{c})}^{2}}{w^{2}}}$$

## 3. Results and Discussion

Through the laser-pulling instrument, glass nanopores with a diameter of 30 to 50 nm were obtained (Figure 1b). As the diameter affects the signal-to-noise ratio of the glass nanopore detection [28], the pore diameter was calculated before each detection by measuring the I-V curve of the nanopore electrolyte and calculating the resistance in the glass nanopore (Figure 1d). Due to the geometric and charge asymmetry of glass nanopores, a significant ion rectification phenomenon can be observed through the I-V curve [29], manifesting as a smaller current magnitude in the negative voltage region compared to the positive voltage region at the same voltage level. This phenomenon may be related to ion accumulation/dissipation [30,31] or electrophoretic capture of mobile ions [32]. The pore diameter of the glass nanopore can be approximately calculated using the following formula [33,34]:

(2) $$R=\frac{\gamma \cot\frac{\theta }{2}}{\pi a}$$

In this case, *R* is the resistance value of the nanopore calculated from the measured I-V curve, *θ* is the tip angle of the conical nanopore (approximately 15° ± 3°), *γ* is the resistivity of the electrolyte, and *a* represents the radius of the nanopore. In theory, during the pulling process, a glass capillary with an inner diameter of 0.7 mm and a length of 7.5 cm will be pulled into a pair of glass nanopores with the same pore diameter. The size of the nanopore is characterized using scanning electron microscopy (SEM), and the exact nanopore diameter can be obtained (Figure 1c). By comparing the calculated and measured values of the glass nanopores pulled from the same glass capillary using both computational and scanning electron microscopy methods, it can be observed that the estimated values were in good agreement with the SEM imaging results, indicating that the computational method can provide accurate measurements of the nanopore diameter.

The glass nanopore was filled with electrolyte solution (1 M KCl, 10 mM Tris-HCl, pH = 8.0) in both the nanopore and the electrochemical cell. A constant voltage was applied to detect the signals generated by the passage of GAD65 and GADAb through the nanopore. When GAD65 or GADAb were added to the electrolyte solution, due to the negative charge carried by the antigen or antibody molecules, the electrophoretic force acting on the protein molecules was opposite to the direction of the electric field. On the other hand, the negatively charged inner wall of the glass nanopore attracts cations in the solution, forming a cation layer on the nanopore wall, with the direction of the electroosmotic flow opposite to the electrophoretic force. In this case, the electrophoretic force is stronger than the electroosmotic flow, resulting in the protein molecules moving in the same direction as the electrophoresis. When the direction of the electric field is from the negative electrode to the positive electrode (represented by a negative voltage), the proteins flow from outside the nanopore into the nanopore (Figure 1a).

**Figure 2:**
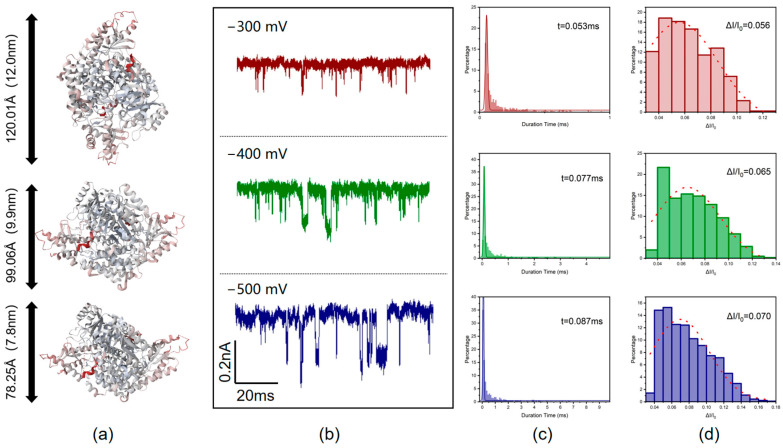
Detection of glutamate decarboxylase molecules using glass nanopores. (**a**) Three-dimensional image of GAD65 protein molecules [35]. (**b**) Current signals are generated by GAD65 molecules passing through glass nanopores under voltages of −300 mV to −500 mV; the signal is rotated 180° for easy observation. (**c**) GAD65 molecule translocation time under voltages of −300 mV to −500 mV. (**d**) Blockade current amplitude ratio ΔI/I_0_ of GAD65 molecules under voltages of −300 mV to −500 mV.

**Figure 3:**
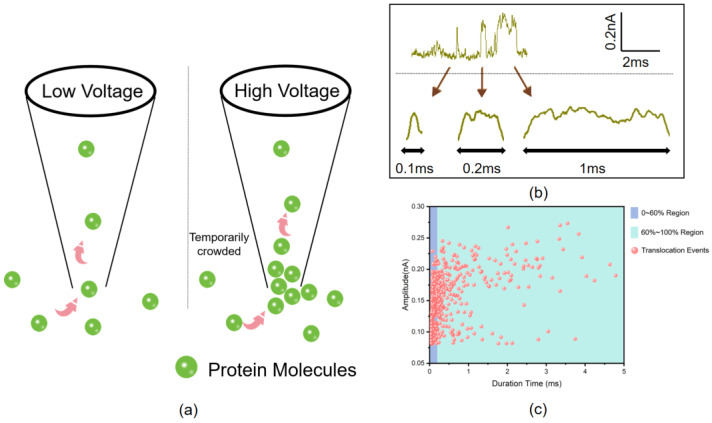
Transient molecular congestion phenomena in nanopores. (**a**) Simultaneous translocation of multiple glutamate decarboxylase molecules through a nanopore. (**b**) Current signatures corresponding to different translocation time events of GAD65 at −400 mV voltage. (**c**) Scatter plot of translocation time versus blockage current amplitude of GAD65 at −400 mV voltage.

The three-dimensional structure of GAD65 shows that the dimension of GAD65 is about 12.0 × 9.9 × 7.8 nm (Figure 2a) [35]. When different voltages were applied across the two ends of the nanopore, proteins translocated through the nanopore, causing changes in the current and generating translocation signals. The translocation signals under different voltages showed different shapes (Figure 2b). As can be seen from the current graphs, when the voltage applied to both ends of the nanopore was increased from −300 mV to −500 mV, both the baseline current and the amplitude of current changes due to molecular translocation increased. It is known that the translocation time of a molecule through a nanopore is related to the length of the molecule, while the ratio of blocked current to baseline current is related to the cross-sectional area of the molecule [36]. In the three voltage conditions tested, all typical translocation signals within 9 s were selected. It was found that the duration time increased with voltage (Figure 2c), which is different from the characteristics of faster translocation speed and shorter translocation time at higher voltages within a low voltage range [37]. This suggests that the effective length of molecules passing through the pore increases. The ratio of blocked current to baseline current ΔI/I_0_ also increased with voltage (Figure 2d), indicating a slight increase in the effective diameter of the molecules passing through the nanopore. One possible reason for this is that at different voltages, molecules pass through the nanopore at different orientations [38]. However, it is also possible that as the voltage increases, the translocation speed of molecules further increases, leading to multiple molecules translocating through the nanopore simultaneously, which causes a temporarily crowded in the nanopore (Figure 3a). This results in some translocation events showing longer duration times and larger blocked currents.

To further validate the above conjecture, taking the potential of −400 mV as an example, the current characteristics corresponding to different duration times are shown in Figure 3b. At −400 mV voltage, events with a duration time of less than 0.2 ms are considered as single-molecule translocation events, while events with a duration time greater than 0.2 ms are considered as multiple-molecule translocation events. The scatter plot of duration time and blocking current amplitude (Figure 3c) shows that events with duration time less than 0.2 ms account for 60% of the total events (the blue-shaded area). To ensure statistical significance, under different voltage conditions, the first 60% and the last 40% of the events are analyzed according to the order of translocation time. The results are summarized in Table 2. It can be seen that when the voltage is continuously increased, the duration time and ΔI/I_0_ of the 0 to 60% events do not change significantly, indicating that the molecules pass through the nanopore as single molecules influenced by the combined effects of electrophoresis and electroosmosis in a rapid manner. However, with further increase in voltage, multiple molecules pass through the nanopore simultaneously and accumulate momentarily at the nanopore, resulting in an increase in the effective molecular diameter inside the nanopore. Therefore, the duration time and ΔI/I_0_ of the 60% to 100% events increase significantly with increasing voltage, which subsequently affects the overall (0 to 100%) values. When the voltage rises to a high voltage region (greater than −600 mV), the increase in voltage has a more significant effect on molecular motion speed than on accumulation at the nanopore, so the translocation time and blocking current ratio decrease with further increase in current.

**Figure 4:**
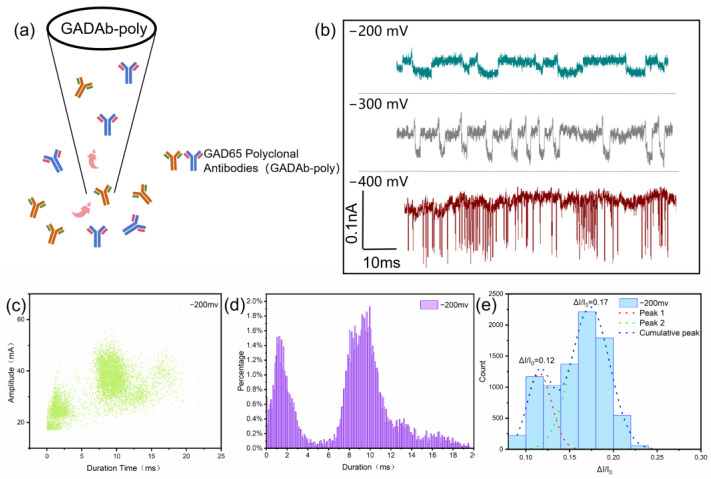
Detection of glutamate decarboxylase polyclonal antibody (GADAb-poly) using glass nanopores. (**a**) Schematic of GADAb-poly translocation through the nanopore. (**b**) Current signals are generated by GADAb-poly passing through glass nanopores under voltages of −200 mV to −400 mV; the signal is rotated 180° for easy observation. (**c**) Scatter plot of translocation time versus blockage current amplitude of GADAb-poly at −200 mV voltage. (**d**) Histograms of translocation time–frequency distribution of GADAb-poly at −200 mV voltage. (**e**) Histograms of ΔI/I_0_ distribution of GADAb-poly at −200 mV voltage.

The antibody produced by a single B cell clone with high homogeneity and only targeting a specific epitope is called a monoclonal antibody. The mixture of antibodies produced by different antibody B cells in the body under the stimulation of antigenic determinants is referred to as polyclonal antibodies [39]. In animals, GADAb may exist in the form of polyclonal antibodies. The glutamate decarboxylase antibody (GADAb) analyzed in the experiment has a Y-type structure similar to other IgG antibodies, but the differences in specific amino acid fragments lead to different surface charges, which further affect the characteristics of specific monoclonal antibodies passing through nanopores. In this case, the glutamate decarboxylase polyclonal antibody (GADAb-poly) was passed through the nanopore (Figure 4a), and the resulting current signals at different voltages were studied (Figure 4b). Under a low voltage of −200 mV, the frequency distribution histogram of translocation times and ΔI/I_0_ in the low voltage region both show two distinct event peaks at 2 ms and 9 ms, 0.12 and 0.17 suggesting the distinct statistical characteristics of polyclonal antibodies in nanopore detection compared to monoclonal antibodies. This provides a way for the practical detection of GADAb in the human body.

**Figure 5:**
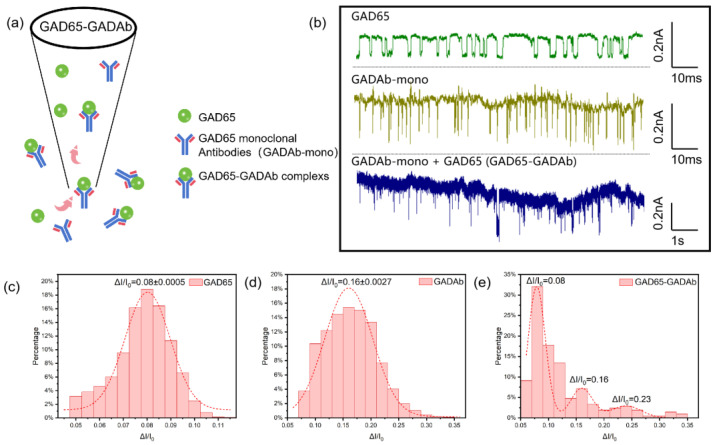
Detection of glutamate decarboxylase (GAD65) and glutamate decarboxylase antibody (GADAb-mono) mixed solutions using glass nanopores. (**a**) Schematic of mixed solution translocation through the nanopore. (**b**) Current signals generated by GAD65, GADAb-mono and their mixture passing through glass nanopores under voltage of 300 mV. (**c**–**e**) Histograms and fitted normal distributions of ΔI/I_0_ at 300 mV voltage for GAD65, GADAb-mono, and the mixed solution of GAD65 and GADAb-mono.

In other studies that use glass nanopores and rely on the specific binding of antigen-antibody interactions for antibody detection, the typical approach involves modifying the antigen molecules on the inner surface of the glass nanopore and then placing the nanopore in an electrolyte solution containing antibodies for detection, the specific binding between antigen-antibody molecules is confirmed through characteristics such as extended translocation time [21,40]. In contrast, our study attempted to premix the antigen-antibody molecules in an electrolyte solution (1 M KCl, 10 mM Tris-HCl, pH = 8.0) and directly detect the mixed solution using unmodified glass nanopores. Using the optimal concentration of antigen or antibody solutions that produce significant current signals in their respective single solutions, GAD65 and GADAb were 1540 pmol/L and 667 pmol/L, respectively. During this part, for clearer results, a monoclonal antibody of GADAb (GADAb-mono) was used, and the two were premixed in a 1:1 volume ratio for 30 min before detection. It was found that at a voltage of 300 mV, the relative current amplitude ratios (ΔI/I_0_) of GAD65 and GADAb solutions were 0.08 and 0.016, respectively (Figure 5c,d). Tests conducted on a mixed solution of GAD65-GADAb also observed these two peaks, while a peak with an amplitude of approximately 0.23, which is approximately equal to the sum of the abscissas of the two peaks, was also observed (Figure 5e). This peak is suspected to correspond to the specific binding of antigen-antibody complexes. Furthermore, a minor peak can be observed at ΔI/I_0_ ≈ 0.32, which is likely representative of the binding of an antibody to a bimolecular antigen complex, demonstrating the bivalent nature of the IgG antibody. Additionally, the fitted peak height in Figure 5e exhibits a good correlation with the antigen and antibody concentrations before mixing. This study achieved simultaneous identification of glutamate decarboxylase (GAD65), glutamate decarboxylase antibody (GADAb), and GAD65-GADAb antigen-antibody complexes in a complex system, providing a new method for early screening of type I diabetes.

## 4. Conclusions

The glass capillary nanopore has the characteristics of low cost, simple manufacturing, high hardness, easy control of size and shape, and easy to be mass-produced. This article has completed the preparation of glass capillary solid-state nanopores with a pore size of 30 nm to 50 nm and applied glass nanopores to the detection and identification of protein molecules and antigen-antibody complexes. The focus is on the detection of glutamate decarboxylase (GAD65) protein, glutamate decarboxylase polyclonal antibodies (GADAb), and their antigen-antibody complexes (GAD65-GADAb). Using GAD65 as a typical protein molecule, this study investigates the significant differences in the translocation time and amplitude ratio of obstructed current between higher voltage ranges (−300 mV to −500 mV) and low voltage ranges (less than −300 mV). Through a proportional division approach, the characteristic events of individual molecules translocating and multiple molecules translocating simultaneously are approximately studied. It is revealed that within this voltage range, as the voltage increases, the temporary accumulation phenomenon inside the nanopore becomes the main factor affecting the overall characteristics of the events. When the voltage is further increased, the effect of voltage on the acceleration of molecular movement is significantly greater than that of pore accumulation, thus reducing the temporary accumulation phenomenon inside the nanopore. In the low voltage range (−200 mV), preliminary identification of the number of antibody species in glutamate decarboxylase polyclonal antibodies (GADAb-poly) is completed, indicating that there are at least two or more monoclonal antibodies. Finally, GAD65 is directly mixed with glutamate decarboxylase monoclonal antibodies (GADAb-mono) and passed through the nanopore without modification or labeling. The identification of three protein binding states in solution is completed, providing a new method for rapid and low-cost detection of GADAb, which is a preliminary screening indicator for autoimmune type I diabetes (immune-mediated).

## Figures and Tables

**Table 1:** Fabrication parameters of glass nanopore.

Heat	Filament	Velocity	Delay	Pull
760	4	29	140	168

**Table 2:** Fitting results of the normal distribution mean for Duration time and ΔI/I_0_ corresponding to GAD65 translocation events with translocation times in the 0–60% and 60–100% ranges, which are ranked by translocation time from shortest to longest.

Grouping of Events	Analysis Type	−300 mV	−400 mV	−500 mV	−600 mV
0–60%	Duration Time	0.052 ms	0.056 ms	0.053 ms	0.056 ms
	ΔI/I_0_	0.051	0.055	0.060	0.060
60–100%	Duration Time	0.12 ms	0.28 ms	0.66 ms	0.29 ms
	ΔI/I_0_	0.081	0.089	0.10	0.090
0–100%	Duration Time	0.053 ms	0.077 ms	0.087 ms	0.086 ms
	ΔI/I_0_	0.056	0.065	0.070	0.066

## Data Availability

The raw data supporting the conclusions of this article will be made available by the authors on request.
